# Porcine Epidemic Diarrhea Virus, Surrogate for Coronavirus Decay Measurement in French Coastal Waters and Contribution to Coronavirus Risk Evaluation

**DOI:** 10.1128/spectrum.01844-23

**Published:** 2023-07-03

**Authors:** Maud Contrant, Lionel Bigault, Mathieu Andraud, Marion Desdouits, Sophie Rocq, Françoise S. Le Guyader, Yannick Blanchard

**Affiliations:** a Viral Genetics and Biosecurity Unit (GVB), French Agency for Food, Environmental and Occupational Health Safety (ANSES), Ploufragan, France; b Epidemiology, Animal Health and Welfare Unit (EPISABE), French Agency for Food, Environmental and Occupational Health Safety (ANSES), Ploufragan, France; c Ifremer, laboratoire de Microbiologie, SG2M/LSEM, BP 21105, Nantes, France; University of Manitoba

**Keywords:** SARS-CoV-2, seawater, half-life, PEDv, environment, surrogate

## Abstract

Severe acute respiratory syndrome coronavirus 2 (SARS-CoV-2) in infected patients mainly displays pulmonary and oronasal tropism; however, the presence of the virus has also been demonstrated in the stools of patients and consequently in wastewater treatment plant effluents, raising the question of the potential risk of environmental contamination (such as seawater contamination) through inadequately treated wastewater spillover into surface or coastal waters even if the environmental detection of viral RNA alone does not substantiate risk of infection. Therefore, here, we decided to experimentally evaluate the persistence of the porcine epidemic diarrhea virus (PEDv), considered as a coronavirus representative model, in the coastal environment of France. Coastal seawater was collected, sterile-filtered, and inoculated with PEDv before incubation for 0 to 4 weeks at four temperatures representative of those measured along the French coasts throughout the year (4, 8, 15, and 24°C). The decay rate of PEDv was determined using mathematical modeling and was used to determine the half-life of the virus along the French coast in accordance with temperatures from 2000 to 2021. We experimentally observed an inverse correlation between seawater temperature and the persistence of infectious viruses in seawater and confirm that the risk of transmission of infectious viruses from contaminated stool in wastewater to seawater during recreational practices is very limited. The present work represents a good model to assess the persistence of coronaviruses in coastal environments and contributes to risk evaluation, not only for SARS-CoV-2 persistence, but also for other coronaviruses, specifically enteric coronaviruses from livestock.

**IMPORTANCE** The present work addresses the question of the persistence of coronavirus in marine environments because SARS-CoV-2 is regularly detected in wastewater treatment plants, and the coastal environment, subjected to increasing anthropogenic pressure and the final receiver of surface waters and sometimes insufficiently depurated wastewater, is particularly at risk. The problem also arises in the possibility of soil contamination by CoV from animals, especially livestock, during manure application, where, by soil impregnation and runoff, these viruses can end up in seawater. Our findings are of interest to researchers and authorities seeking to monitor coronaviruses in the environment, either in tourist areas or in regions of the world where centralized systems for wastewater treatment are not implemented, and more broadly, to the scientific community involved in “One Health” approaches.

## INTRODUCTION

The emergence of the human coronavirus, severe acute respiratory syndrome coronavirus 2 (SARS-CoV-2), accompanied by its worldwide spread leading to the COVID pandemic (671 million cases and 6.85 million deaths on February 2023; WHO [World Health Organization]), reminds us, if needed, of the health hazard posed by coronaviruses.

Many coronaviruses exist and are associated with diverse tropisms (respiratory for the majority of human coronaviruses [1–3], enteric for a large number of livestock CoVs [4, 5], and neurological in some cases [e.g., porcine hemagglutinating encephalomyelitis virus] [6]). Interestingly, SARS-CoV-2, in addition to its respiratory tropism, has been repeatedly detected in stool samples of infected patients (7) (for review, see reference 8) for long periods (9), even in the absence of any gastrointestinal symptoms (10, 11), calling into question, in the early time of the pandemic, the potential risk of fecal-oral or fecal-respiratory transmission (12–18). Reports on the isolation of infectious SARS-CoV-2 from the feces and urine of COVID-19 patients have been documented but remain rare (reviewed in reference 19). Interestingly, the SARS-CoV-2 genome has been detected in raw wastewater from different metropolitan areas, including Paris (20), with concentrations correlating with the estimated number of COVID-19 cases. The genome has also been detected in treated effluents from sewage treatment plants, but to a lesser extent, suggesting that SARS-CoV-2 may contaminate the environment through accidental or direct wastewater discharges (21–23). SARS-CoV-2 RNA was found to be significantly more persistent than infectious SARS-CoV-2, indicating that environmental detection of RNA alone does not substantiate the risk of infection (19, 24). The transmission of enteric viruses through recreational use of sewage-contaminated water is well documented (25, 26). The resistance of virions to harsh conditions is highly variable, but coronaviruses are considered fairly resistant in the environment compared to other viral families (for review, see references 27, 28). Therefore, even if the infectious capacity of SARS-CoV-2 from feces is very limited (19, 24), transmission through wastewater-contaminated waters is theoretically possible as is environmental contamination with enteric coronaviruses, including livestock CoV following the spread of manures.

Previous studies on other CoVs (such as SARS-CoV, transmissible gastroenteritis virus [TGEV], and murine hepatitis virus [MHV]) have shown them to be detectable in sewage for 2 to 4 days, in tap water for up to 10 days at 23 to 25°C, and up to 100 days at 4°C (for review, see reference 29), but studies on the presence and persistence of CoVs in seawater remain scarce. One study showed that SARS-CoV-2 lost its infectivity within 2 days in seawater at 23°C (30), but half-lives were not calculated, and this temperature is higher than those encountered in North-Western European countries like France. Another study calculated a time, in days, for virus titres to decrease by 90% (T90) of 1.1 day in filtered seawater at 20°C for the porcine respiratory coronavirus (PRCV) (31), and finally, a study showed a high impact of the temperature on the decay of SARS-CoV-2 in seawater by comparing conditions at 20°C and 4°C, with a T90 of 7 to 10 days (32). Intermediate temperatures were not tested. The possible long survival in water systems raises concerns about the persistence of CoVs, including SARS-CoV-2, in the coastal environment. Therefore, risk assessment of viral exposure and transmission requires additional experimental evidence of CoV persistence in seawater.

To evaluate the stability and resistance of CoVs and by extension of SARS-CoV-2 in the coastal environment, the genomic and infectious titers, the latter directly correlated to the infection risk, of the strain CV777 of porcine epidemic diarrhea virus (PEDv) (an alpha-CoV), as model and surrogate, were measured in natural coastal seawater for 28 days. Then, the results were used to set up a mathematical model of coronavirus decay in seawater at a range of temperatures (8°C, 15°C, and 24°C) representative of the annual variation of the French coastal waters. A fourth temperature of 4°C was also investigated as a reference temperature, allowing for comparisons with other studies addressing decay in different types of water. The decay rate of PEDv was then used to determine the half-life of the virus in French coastal waters, using the temperatures reported for each trimester (year quarters) from 2000 to 2021.

## RESULTS

### Survey of PEDv decay in seawater.

The half-life of the coronavirus in seawater was poorly characterized when starting these experiments. Therefore, in a preliminary experiment (data not show), we measured the decay of virions at four different temperatures (4, 8, 15, and 24°C) for 16 days. At the two lowest temperatures, we observed only a slight decrease in infection of 5% and 12% at 4°C and 8°C, respectively, by day 16. At day 16, for the 15°C temperature, the infectious titer dropped by about 40% (from 6 log10 50% tissue culture infective dose [TCID_50_] to 3.6 log10 TCID_50_), and complete loss of infection was observed after 7 days at 24°C (0.8 log10 TCID_50_). In parallel with infectivity, a measure of genomic load was performed using reverse transcriptase quantitative PCR (RT-qPCR), which revealed the stability of the viral genome throughout the 16 days duration of this preliminary experiment. Therefore, in order to be able to decipher more precisely the viral decay of PEDv in seawater, three independent series of experiments were performed with the same temperature settings (4, 8, 15, and 24°C) but extended to 28 days.

**FIG 1:**
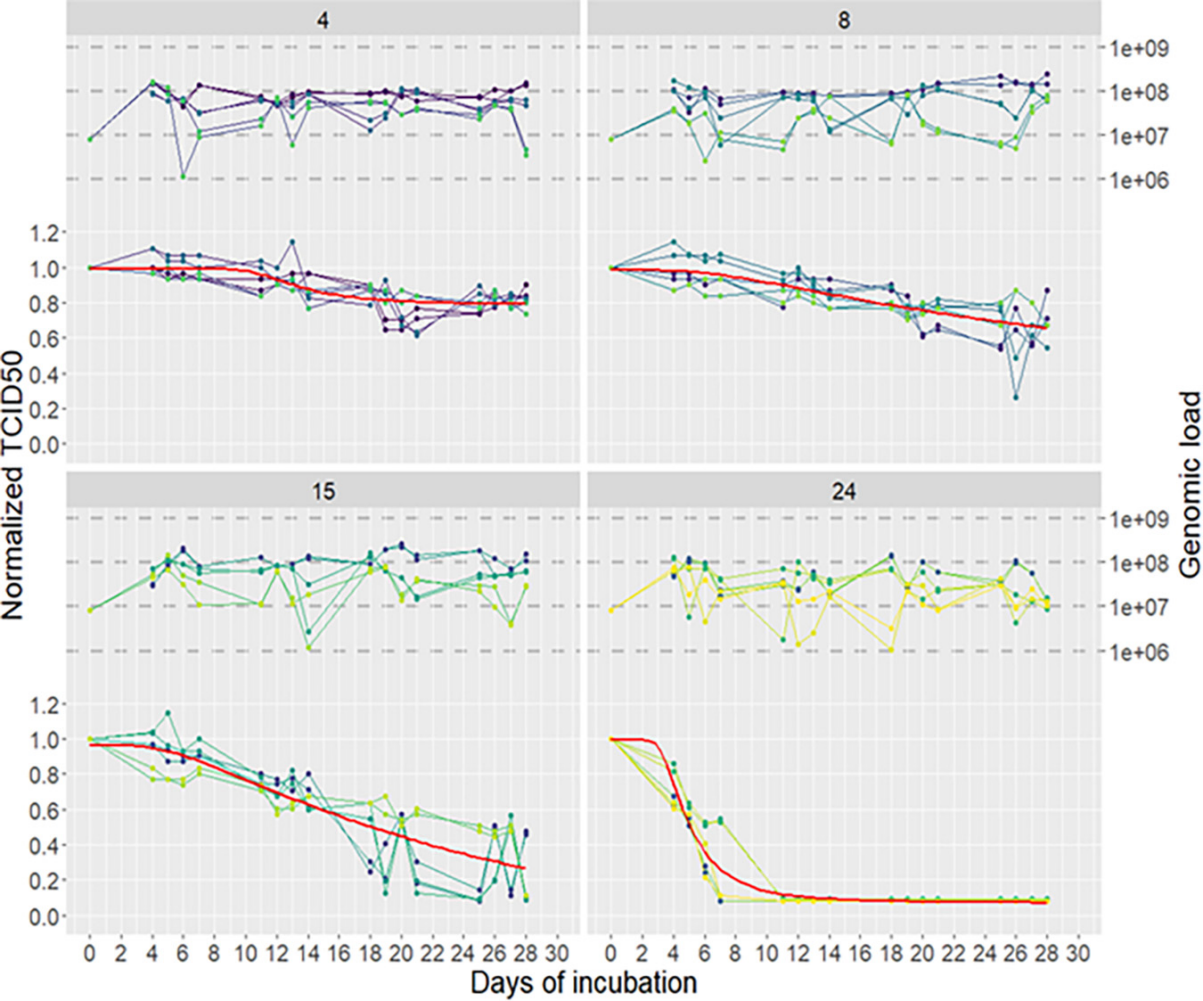
Genomic and infectious titers of PEDv incubated in seawater at four different temperatures for 28 days. One-milliliter aliquots of seawater were spiked with the PEDv CV777 virus stock to achieve a load of 6 log10 (1 × 10^6^) TCID_50_/mL and 1 × 10^8^ genome copy/mL then incubated in water baths for 28 days at 4 different temperatures: 4°C, upper left panel; 8°C, upper right panel; 15°C, lower left panel; and 24°C, lower right panel. The genomic load (genome copies, in log scale, right axis, upper part of each panel) and infectious titer (ratio of the TCID_50_ measured at each time point [days of incubation, horizontal axis] on the one at day 0 for each experiment, left axis, bottom part of each panel) were measured 4 days a week (D4 to D7, D11 to D14, D18 to D21, and D25 to D28) by RT-qPCR and IPMA, respectively, in duplicate for each aliquot on the same aliquot and during three independent experiments. For each condition, the 6 measures are depicted with blue to yellow points and lines. The Weibull models calculated for the infectious titer decay are represented by the red curves.

Table 1 gives the mean TCID_50_ results of the 3 series of experiments, and Fig. 1 shows the daily evolution of TCID_50_, normalized to the initial (*t*_0_) TCID_50_ for 28 days at the four different temperatures.

**Table 1:** Log10 TCID_50_ and percentage from TCID_50_ at D0 of PEDv for viral titer quantification results during incubation in seawater at 4°C, 8°C, 18°C, and 24°C, per day, during 28 days^*a*^

DPI	Log10 TCID_50_ (standard deviation) according to temp											
	4°C			8°C			15°C			24°C		
	Mean	%	SD	Mean	%	SD	Mean	%	SD	Mean	%	SD
0	5.97	100.00	0.30	5.97	100.00	0.30	5.99	100	0.28	5.94	100	0.33
4	6.10	102.18	0.18	5.80	97.15	0.41	5.57	92.98	0.55	4.17	70.20	0.45
5	5.90	98.83	0.22	5.80	97.15	0.24	5.40	90.15	0.62	3.43	57.74	0.16
6	5.83	97.65	0.16	5.67	94.97	0.34	5.10	85.14	0.42	2.13	35.85	0.72
7	5.80	97.15	0.21	5.70	95.48	0.31	5.33	88.98	0.29	1.43	24.07	1.22
11	5.47	91.62	0.29	5.20	87.10	0.24	4.47	74.62	0.29	0.50	8.41	0.00
12	5.53	92.63	0.15	5.50	92.13	0.31	4.03	67.27	0.53	0.50	8.41	0.00
13	5.67	94.97	0.39	5.23	87.60	0.30	4.27	71.28	0.46	0.50	8.41	0.00
14	5.23	87.60	0.64	5.00	83.75	0.47	4.07	67.94	0.60	0.50	8.41	0.00
18	5.20	87.10	0.41	5.00	83.75	0.43	2.97	49.58	1.03	0.50	8.41	0.00
19	4.77	79.90	0.50	4.50	75.38	0.36	2.20	36.72	1.39	0.50	8.41	0.00
20	4.40	73.70	0.59	4.27	71.52	0.43	3.20	53.42	0.21	0.50	8.41	0.00
21	4.40	73.70	0.73	4.43	74.20	0.27	2.00	33.38	1.30	0.50	8.41	0.00
25	4.77	79.90	0.21	4.07	68.17	0.59	1.40	23.37	1.25	0.50	8.41	0.00
26	4.93	82.58	0.37	3.93	65.83	1.54	2.33	38.89	0.97	0.50	8.41	0.00
27	4.83	80.90	0.24	4.00	67.00	0.70	2.27	37.89	1.14	0.50	8.41	0.00
28	4.90	82.08	0.42	4.13	69.18	1.37	1.19	22.87	0.50	0.00	0.00	0.00

a One-milliliter aliquots of water were spiked with the CV777 virus stock to achieve a load of 6 log10 (1.10e6) TCID_50_/mL (6 log10 TCID_50_) and then incubated in water baths at 4 different temperatures for 28 days. Infectious titers were determined at days 0, 4 to 7, 11 to 14, 18 to 21, and 25 to 28 by IPMA. DPI, day post infection.

We observed good repeatability for the samples collected at different time points in the experiment, with the same trend for each of the triplicates according to the temperature. However, significant variability between the triplicates on days 20 to 28 at a temperature of 15°C was also observed (day 25; 0.5 log10 TCID_50_ for series 1 and 2 and 2.9 log10 TCID_50_ for series 3), which gives a random and blurred aspect to this region of the curve. The complete experiment, over 4 weeks, confirmed the trend observed during the first test. At 4°C and 8°C, we observed an overall good stability of the PEDv infectious titer with 82% and 69% of the initial TCID_50_ value maintained after 28 days of incubation in our coastal water sample, respectively. At 15°C, 88% of the TCID_50_ was maintained during the first 7 days, and then the infectious titer dropped to 67% of the initial TCID_50_ during the next 7 days followed by a regular decline of the TCID_50_ value to 23% of the initial TCID_50_ by day 28. In the last part of the experiment, the individual TCID_50_ values at 15°C were much more dispersed, as reflected by the increase in the standard deviation values for the last points from day 18 onward. Finally, the complete loss of infectious PEDv after 7 days at 24°C was confirmed. In parallel with the measurement of the infectious titer, the viral genome concentration was measured by RT-qPCR to ascertain the presence of viral particles in the incubation medium used for the infection (Fig. 1; see also the supplemental material). Despite day-to-day variations, on the 28-day duration of the experiment, the PEDv genome loads remained stable for the four experimental conditions, confirming that the decrease in infectious titer was not due to the loss of virus when preparing the viral inoculum for TCID_50_ experiments, but to the time- and temperature-dependent degradation of the viral particles.

### Modeling the infectious load decay of coronavirus in coastal water.

Two models, a biexponential model and a Weibull-type model (33, 34), were evaluated to define a mathematical model of the decrease in PEDv viral titers in coastal water over time as a function of temperature. Akaike's information criterion was lower with the Weibull model than with the biexponential model (−728 and −634, respectively), indicating a better fit of the data with the Weibull model. The model parameters, representing the average kinetics estimated at the population level are listed in Table 2.

**Table 2:** Parameters estimated at the population level representing the average kinetics for the biexponential and the Weibull model

Parameter	Biexponential model parameters (standard deviation)				Weibull model (standard deviation)		
	a_1_	δ_1_	a_2_	δ_2_	D	δ	α
Fixed effect	0.88 (0.11)	0.003 (0.001)	0.16 (0.02)	0.06 (0.08)	0.71 (0.09)	0.02 (0.002)	0.48 (0.09)
Temperature covariate	0.006 (0.005)	0.17^*a*^ (0.02)	−0.03 (0.05)	−0.03 (0.11)	0.01^*a*^ (0.005)	0.1^*a*^ (0.007)	0.08^*a*^ (0.01)
AIC	−638				−737		

a *P* value < 0.05.

**FIG 2:**
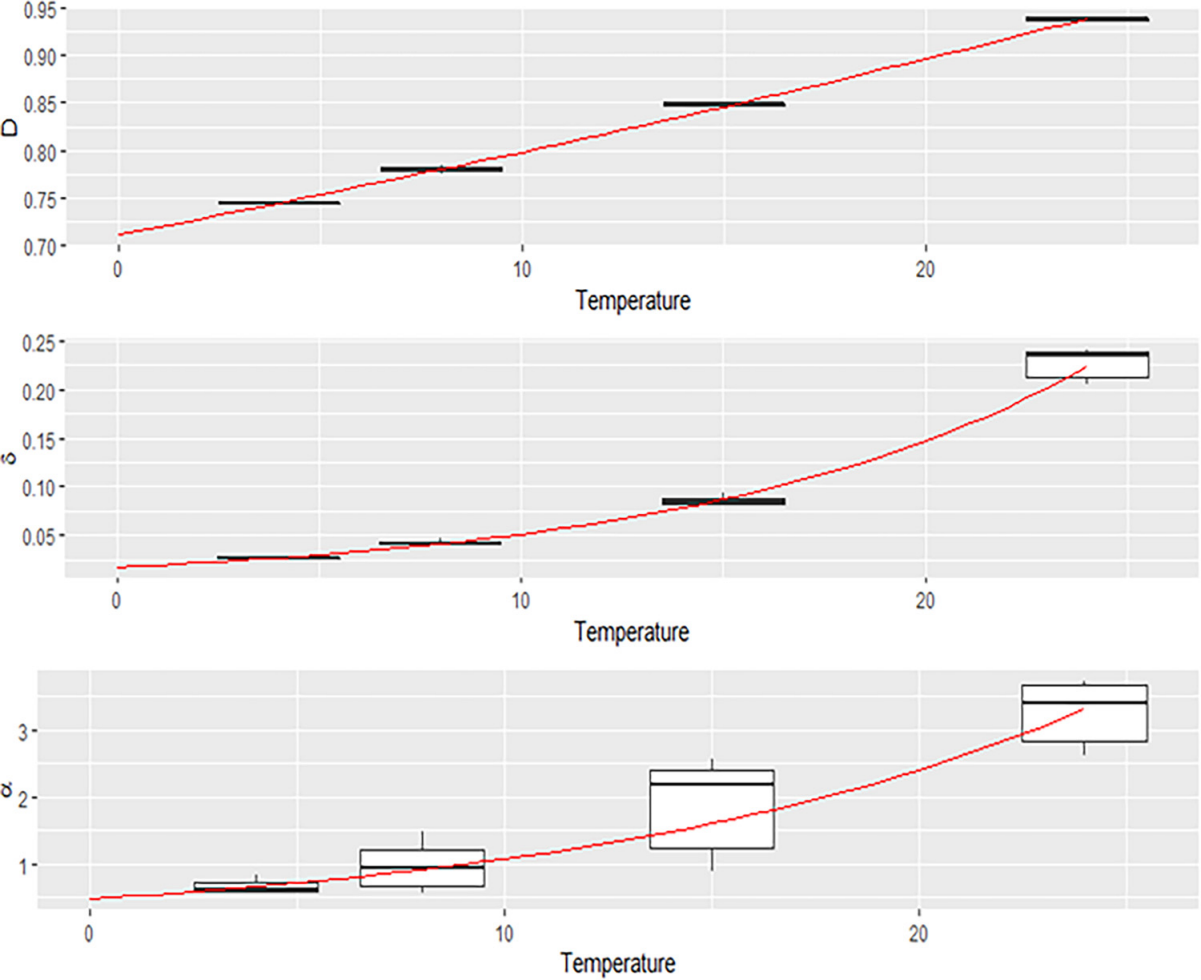
Correlation between the parameters estimated for each kinetic and the water temperature.

The log-linear regression model revealed a significant effect of temperature on all of the individual parameters, accounting for interindividual variability. Correlation between the parameters estimated for each kinetic and the water temperature is shown in Fig. 2. The parameters governing the decrease (δ and α) reflect the persistence observed at 4°C over the duration of the experiment. These parameters showed a strong dependence on the water temperature. A strong exponential increase in δ was observed between 15°C and 24°C, indicating an impact on the initial decrease. The increase in parameter α, which governs the speed of convergence toward *V*_∞ (asymptotic viral load), also showed an exponential trend, varying from 1 to 3 between 4 and 24°C.

### Analysis of the persistence of coronaviruses along the French seashore.

We applied the coronavirus decay model along the French seashore, considering the variations in temperature occurring within a year.

**FIG 3:**
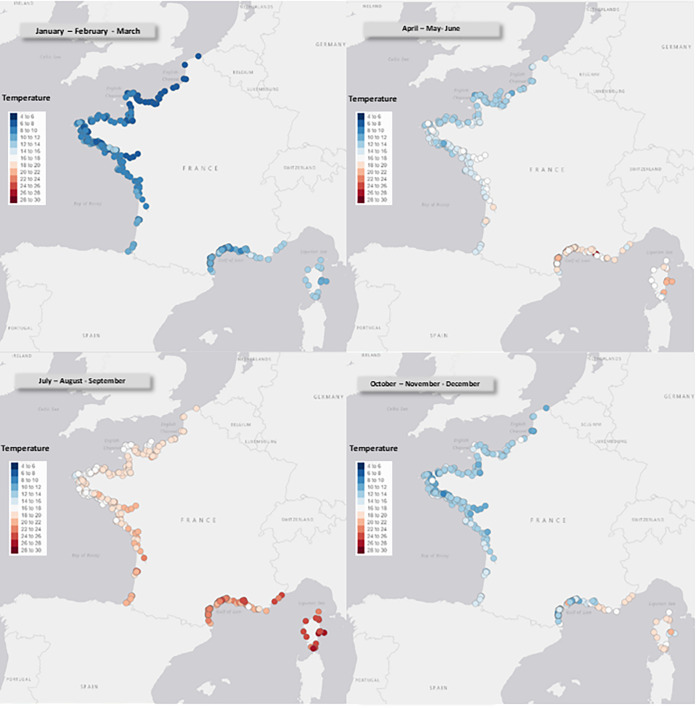
Mean quarterly temperatures of surface waters on the French coast from 2000 to 2021.

The quarterly average temperature varied seasonally, ranging from 6°C to 14°C in winter and from 14 to 26°C in summer. The map displayed in Fig. 3 highlights geographical differences between northern and southern France as well as between the Mediterranean coast and the Atlantic Ocean in the west.

**FIG 4:**
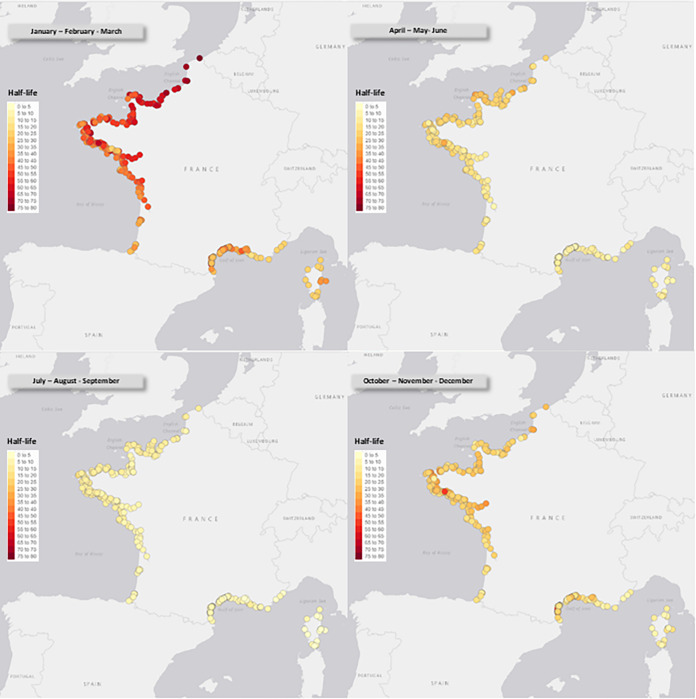
Estimated half-life (in days) of coronavirus in surface water, based on quarterly average temperature for each collection location.

The half-life of the virus in the surface water at each sampling point was evaluated based on the quarterly average temperature and is represented as a heat map in Fig. 4. Reflecting the analysis of the temperature records, a huge seasonal effect was observed, with a half-life of coronavirus infectivity of up to 70 to 80 days during the coldest period, falling below 20 days at all locations during the summer. The northern area exhibited the highest half-life seasonal variation compared to that of Corsica, where water temperatures remained much more stable throughout the year.

## DISCUSSION

Viral contamination of water or food by human sewage is a long-documented origin of gastroenteritis outbreaks, as exemplified by the contamination of oysters by human norovirus (HuNoV) (35, 36). At the beginning of the COVID epidemic, the question regarding SARS-CoV-2 risk of exposure and transmission to human during recreational activities was highlighted. CoVs are resistant viruses, and SARS-CoV-2 virions remained infectious for up to 3 h in aerosols and for 3 days on artificial surfaces, respectively. SARS-CoV-2 virions are also stable over a wide range of pH values at room temperature (pH 3 to 10) (37–44). A few studies have concluded that the risk of infection following exposure to SARS-CoV-2 in water is low (19, 24, 28). However, the evaluation of CoV survival in water and especially in seawater will be informative for SARS-CoV-2 but also for other CoVs, especially enteric CoV.

This study aimed to monitor experimentally the survival/persistence of PEDv as a representative of CoV and also as a surrogate for SARS-CoV-2 in marine waters.

Coronaviruses (order, *Nidovirales*; suborder, *Cornidovirinae*; family, *Coronaviridae*; subfamily, *Orthocoronavirinae*) show very strong genetic diversity and a high prevalence in nature (45). This family of viruses primarily infects a wide host spectrum of mammals and birds (46). Based on the variety of nonstructural accessory proteins, antigenic properties, and host ranges, four genera of CoVs have been described (alpha-, beta-, delta-, and gamma-CoV), among which alpha- and beta-CoVs include viruses infecting humans (HCoV). Although coronaviruses are divided into four distinct genera, they all share similar physicochemical properties with an enveloped spherical viral particle of 120 nm diameter and the same genomic organization with a single-stranded RNA genome of positive polarity (25 to 30 kb). SARS-CoV-2 (a beta-CoV) like some other coronaviruses (SARS-CoV, Middle East respiratory syndrome [MERS]) is zoonotic, leading to its classification as a biosafety level 3 (BSL3)-like pathogen with a commitment to need for an L3 laboratory facility for its manipulation. The number of BSL3 laboratories is much more limited than that of BSL2 laboratories, and nonzoonotic coronaviruses have been largely and successfully used as surrogate of SARS-CoV-2 for studies in BSL2 facilities but present some limitations.

For example, nonenveloped viruses, such as bacteriophage MS2, pose problems of misinterpretation and accuracy and are known to be more resistant than enveloped viruses (42, 43). The use of a coronavirus as a surrogate provides greater reliability, as coronaviruses form an organizational unit with similar biophysical properties and genomic structures. Moreover, studies (34) have shown a similar temperature and relative humidity persistence potential of different coronaviruses infecting human and nonhuman mammals on surfaces and in suspension, confirming the potential of animal coronaviruses as surrogates.

For this reason, we previously used porcine epidemic diarrhea virus (PEDv) and infectious bronchitis virus (IBV), both without any zoonotic potential, as viral substitutes for SARS-CoV-2 (47) and found that treatment in a climate chamber at 70°C for 1 h with a 75% humidity rate was adequate for enabling substantial decontamination of both model animal coronaviruses, confirming for these two viruses the overall inactivation properties shared by coronaviruses (34). In the current study, we relied on the CV777 strain of PEDv, a swine alphacoronavirus, as a representative model of enteric CoV and as a surrogate for SARS-CoV-2.

Recently, the PEDv has been used several times, as surrogate for HCoV, including SARS-CoV-2, for the following: (i) to compare analytical methods to detect SARS-CoV-2 in wastewater (48), (ii) as a process control for the monitoring of the occurrence of SARS-CoV-2 in 2020 in six wastewater treatments plants in Spain (49), (iii) to evaluate the use of viability RT-qPCR for the selective discrimination and surveillance of infectious SARS-CoV-2 in secondary treated wastewater (50), (iv) to test the temperature sensitivity of different CoV in fomites (51), (v) to evaluate the effects of dry- and moist-air controlled heating treatment on structure and chemical integrity, decontamination yield, and filtration performance of surgical masks and FFP2 respirators (47), and (vi) to test inactivation of PEDv on contaminated surgery masks by low-concentrated sodium hypochlorite dispersion (52). Furthermore, in a previous study, we demonstrated and quantified the bioaccumulation of PEDv or inactivated SARS-CoV-2 in oysters and observed a similar tissue distribution and efficacy of bioaccumulation between the two coronaviruses, suggesting that PEDv is an adequate surrogate for SARS-CoV-2 in marine environments (53).

Various studies using surrogate viruses (32) and addressing the detection and survival of coronaviruses in wastewater, river water, or filtered or sterilized water have been conducted over the past 2 years (34, 49, 54). Others used both surrogates and the SARS-CoV-2 (32, 33) but were conducted either at low temperatures (4°C) or at higher temperatures (20 to 25°C). Yet, the temperature gradient was not considered in the same model. The synthesis that can be drawn from these studies shows a common trend, in which different viruses remain infectious for several hours to several weeks, depending on the experiment, in different types of water (pure water, surface water, seawater, and sterilized wastewater), with infectivity maintained for a longer period of time at the lowest temperatures (30, 39, 55). Recently, a study (56) showed an inverse correlation between temperature and viral titer for the viability of SARS-CoV-2 in media and artificial seawater. These studies illustrate that intact coronavirus particles present in coastal waters may remain infectious for some time.

Our results present a follow-up of the decay of the virus over a long period with a wide temperature gradient (from 4°C to 24°C), similar to the temperature gradient observed in French coastal waters throughout the year, associated with a mathematical model used for evaluating the virus half-life along the French shore throughout the year. This experiment clearly shows that seawater temperature has a dramatic effect on the duration of the infectious capacity of PEDv and, by homology, on coronaviruses including SARS-CoV-2. The parallel evaluation of the genomic load of the samples throughout the experiment precludes the possible bias of a reduction in the viral load due to artifacts such as adsorption on the tube walls or other phenomena. Under summer conditions representative of Mediterranean conditions (24°C), we observed a rapid loss of the infectious capacity of the virus in seawater, with a loss of almost 2 log10 TCID_50_ at 3 to 4 days and a total loss at day 7. Under conditions mimicking the spring Atlantic coast (~15°C), the effect was more gradual, with a loss of 1 log10 in 1 week and almost total loss at 3 weeks. In winter conditions (4°C and 8°C), the infectious virus is more stable, with survival extending beyond 4 weeks.

In another study assessing SARS-CoV-2 survival in cell culture medium, with a similar range of temperatures as ours (4°C, 13°C, 21°C, and 25°C), the best virus survival was also observed with the coolest conditions; the virus was relatively stable for all the temperature conditions with the maximum log10 reduction of 1.17 virus titer at 4 days postcontamination at 25°C (4.7 log10 TCID_50_ starting dose) (41). Coronaviruses are sensitive to extremely acidic and basic pH. Therefore, the faster inactivation of PEDv in seawater can be explained by the high salinity concentration in combination with alkaline conditions (pH > 8) in seawater compared to that in culture medium (41), wastewater, and pure water (38).

Using the experimental kinetics of the viral decay of PEDv as a function of real temperature data along the French coast during the year (considering the mean temperatures reported from 2000 to 2021) allowed us to evaluate the half-life of the virus according to seasons. The risk of viral transmission is correlated with a longer half-life of the virus and a higher frequentation of human populations in coastal environments. Fortunately, these two parameters are inversely correlated with a peak of human population in coastal areas during summer, when the half-life of the virus is the shortest, owing to the higher water temperature which is deleterious to the virus. Conversely, the better survival conditions for the virus in winter is balanced by a lower population size and frequentation in the recreational usage of coastal areas. The situation is somewhat different concerning workers, such as for oyster farmers or fisherman, in contact with water all year even at the coldest temperature when virus half-life is the longest. In our previous study, we showed a low bioaccumulation efficiency of SARS-CoV-2 in oysters, as well as the absence of detection of this virus in seawater and shellfish samples collected on the French coast from April to August 2020 (53), confirming that the actual risk of contamination in food such as shellfish by SARS-CoV-2 is low. Furthermore, if we consider the effect of tides, bringing an additional physical dispersion and dilution effect on the virus, all of these parameters favor a low risk of coronavirus contamination during seashore activities.

It is important to mention that the effect of seawater on the decay of PEDv might be underestimated under our experimental conditions, considering the action of additional parameters of seawater, such as the variation in salinity (this study used only one salinity representative of the Brittany coast), the effect of solar radiation, a known driver of viral decay with summer exhibiting higher UV radiation exposure than winter and the microbiota present in seawater, which has a significant influence on virus survival (32, 57).

Given the low survival of SARS-CoV-2 in sewage (28), the main risk of SARS-CoV-2 contamination through coastal water exposure likely lies in the direct release of raw sewage, places without connection to wastewater collection systems, ports, and in the event of a sanitation accident or human feces in seawater. The question also arises of soil contamination during spreading by slurry potentially contaminated with coronaviruses from livestock (for example, PEDv, responsible for large epidemics and having a significant impact on the pork industry [58]) and therefore by runoff and water contamination. This work could thus be used for the identification of areas that are the most at risk for humans in the case of spillover before treatment, as well as for the identification of maritime areas at risk of virus transmission to the marine animal population and, therefore, to be monitored as a priority. Indeed, as cases of SARS-CoV-2 contamination have been reported in various terrestrial animal species (59–62), it might also infect marine mammals (63). Alpha and gammacoronavirus infections are already known to cause respiratory diseases in aquatic mammals, and recent studies have shown that several species of marine mammals possessed the SARS-CoV-2 receptor, ACE2 (64, 65), with amino acid sequences highly conserved between human and marine mammal species. The binding of SARS-CoV-2 to ACE2 is an essential step in the infection of SARS-CoV-2, which can therefore make these animals susceptible to SARS-CoV-2 infection (66, 67). Thus, contamination of seawater from sewage, wastewater effluent, or urban and agricultural runoff and the survival of SARS-CoV-2 are potentially a risk for marine mammals. Guo et al. modeled this risk by analyzing the possible dispersion of the virus following contamination in seawater and confirmed that this risk of infection is directly linked to viral concentrations but also suggested a critical role of the temperature of the water (68).

## MATERIALS AND METHODS

### Cells and virus.

Vero cells (ATCC CCL-81) are maintained in Eagle's minimum essential medium (EMEM) (Thermo Fisher Scientific, France) supplemented with 10% fetal calf serum (reference 702774; Corning) and 1% penicillin/streptomycin (reference 11548876; Gibco).

CV777 viral strain (GenBank accession number AF353511) of porcine epidemic diarrhea virus (PEDv) was used as a surrogate of the SARS-CoV-2. Briefly, the virus is amplified on Vero cells, in an infection medium, composed of EMEM supplemented with 0.3% tryptone phosphate broth, 0.02% yeast extract, 1% penicillin/streptomycin, and 10 μg/mL trypsin (reference 215240; Difco). After 16 h of infection, the cells are lysed by three successive freezing (−80°C) and thawing (37°C) cycles. The culture medium is clarified by rapid centrifugation at 10,000 × g for 10 min, and then the virus is pelletized for 4 h at 20,000 × g and taken up in 1/100 of phosphate-buffered saline (PBS) of the initial volume of the CV777 inoculum. Viral titer was determined by immunoperoxidase monolayer assay (IPMA) (detection limit, 0.5 TCID_50_/mL) and genomic titer by one-step RT-qPCR (detection limit, 50 copies/5 μL of extract) (69). The viral stock was titrated at 1.7 × 10e8 TCID_50_/mL.

### Determination of the TCID_50_ by immunoperoxidase monolayer assay.

In a 96-well plate, 8 × 10^4^ Vero cells are seeded per well and allowed to adhere for at least 6 h. The cells are washed 3 times with PBS (Sigma, France) and then infected with 100 μL of virus diluted in the infection medium. The infection is stopped after 16 h and the cells fixed with 50 μL of 80% acetone for 20 min at −20°C. After drying for 30 min at room temperature (RT), the endogenous peroxidases are neutralized with 50 μL of a solution of 99% methanol/1% H_2_O_2_ for 30 min at RT. Wells are washed twice with 200 μL of PBS Tween (PBST) (Sigma; no. P3563) and then blocked for 90 min at 37°C with a solution of PBST + 5% milk (PBST5) (Dutscher, no. 2516188). The presence of viral proteins is detected by incubation with 100 μL of an anti-PEDv pig serum diluted to 1:300 in PBST5 for 1 h at 37°C, followed by 3 washes of 200 μL PBST, and then 1 h of incubation at 37°C in the presence of 100 μL of Goat anti-pig IgG coupled to horseradish peroxidase (HRP) (Sigma, France; no. AP166P) diluted to 1/300 in PBST5. After three washes in PBST, the visualization is made by adding 50 μL of AEC/H_2_O_2_ (3-amino-9-ethylcarbazole; Sigma; no. A6926) as developer for 10 min RT. The reaction is stopped by removing the developer followed by a last wash with PBS. The viral titer is determined by the Kärber method. For analysis, log of TCID_50_ for each point are normalized as the ratio against the initial log of TCID_50_. TCID_50_ sensitivity is as low as 0.5 × 10e1 TCID_50_, which is therefore the limit of detection.

### Viral decay trial.

The impact of seawater temperature on the stability and survival of PEDv, chosen as a substitute model for SARS-CoV-2, is evaluated in natural coastal seawater collected and sand-filtered on 21 October 2020 at an experimental oyster farm on the French Atlantic coast (PMMB, Ifremer, Bouin, France), where it is used for growing oysters. At sampling, pH was 8.71, salinity 33.9, and turbidity 2.7 nephelometric turbidity units (NTU). The seawater was then aliquoted and kept frozen at −80°C for 3 to 8 months before being used. The filtered coastal seawater was spiked with the CV777 virus stock to achieve a load of 1.10^6^ TCID_50_/mL and 1.10^8^ genome copies/mL. To prevent any temperature variations due to sample manipulation, as well as the risk of contamination, 1-mL aliquots of this spiked water were incubated in water baths at 4°C, 8°C, 15°C, and 24°C, in the dark, throughout the experiments. A preliminary experiment lasted 16 days with aliquots that were taken at random from day 0 to day 3, day 7 to day 10, and day 14 to day 16. Then, based on these first results, three series of experiments were conducted during 28 days with aliquots randomly sampled on day 4 to day 7, day 11 to day 14, day 18 to day 21, and day 25 to day 28. For each time/temperature pair studied, aliquots were analyzed by PEDv-specific RT-qPCR to quantify the viral genome and by TCID_50_ to determine the infectious capacity of the virus. The excess spiked seawater on day 0 was stored at −80°C for subsequent genomic load determination (69) and TCID_50_ to precisely quantify and check the input.

### Viral decay modeling.

The objective here is to define a mathematical model representing the decrease in PEDv viral titers over time as a function of temperature. Two models were selected among those recently used for similar analysis, a biexponential model and a Weibull-type model (33, 34). Data were normalized for TCID_50_, each point (_n_TCID_50_) treated as the ratio between the value obtained at point *t*_n_ and the initial value at point *t*_0_.

### (i) The biexponential model.

This model is expressed as a function of 4 parameters, two threshold values a_1 and a_2 and two decay rates δ_1 and δ_2:

$$V(t)=a_{1}\;\exp(-\delta_{1}\;t)\;+\;a_{2}\;\exp(-\delta_{2}\;t)$$

Thus, the initial titer is expressed by *V*(0) = a_1 + a_2, to which two successive exponential decreasing phases.

### (ii) The Weibull model.

This model is also based on 4 parameters

$$V(t)=1-D\;\text{*}\exp(-{\left(\delta t\right)}^{-\alpha })\;$$

In this model, an asymptotic decrease is represented towards a threshold value *V*_∞_ = 1 − *D*. The parameter δ governs the initial phase of decay when the parameter α influences the behavior over longer durations and the speed of convergence towards the value *V*_∞_.

### (iii) Parameter estimation.

The data used to estimate the parameters are shown in Fig. 1 and Table 1. Each panel corresponds to a water temperature (4, 8, 15, and 24°C); for each of them, six kinetics were considered (tree separate experiments analyzed on duplicate). The estimation of the parameters was carried out using a nonlinear mixed-effect model for which the kinetics of the repetitions are considered as a longitudinal follow-up in a population. Briefly, for each model, the parameters θ are estimated at the population level. The individual parameters were assumed to be log-normally distributed, thus ensuring their positivity. The parameters of individual *i* are given by

log(θ ^ i)=log(θ ^ pop) + β_Temp * Temp + η_(θ ^ i)

where θ_pop represents the median parameter independent of temperature at the population level. η_(θ^*i*) are random effects vectors assumed to be independent centered Gaussian vectors with variance ω_θ^2, representing interindividual variability. The temperature is integrated as a covariate for all of the parameters and has an impact when the associated parameter β_Temp is significantly different from 0. The quality of adjustment of the models is evaluated according to the Akaike criterion (AIC), which is a measure of the relative quality of a statistical model for a given set of data, the model having the lowest AIC being selected (70).

### (iv) Seawater temperature along the French Coast.

The temperature of the water is regularly monitored on different site of the seashore in France. In this study, data collected in continental French territory were considered. The 308 sampling points were included for which temperature was recorded between 2000 and 2021 at different depths (71). Here, we focused on surface waters only, with a depth varying between 0 and 1 m. The dataset consisted in 54,000 temperature records, which were analyzed quarterly from January to December to derive the average quarter temperature for each year. Feeding the decay model with these temperature records allowed for evaluating the virus half-life for each sampling location and each quarter.
